# Cardiac Shock Wave Therapy Ameliorates Myocardial Ischemia in Patients With Chronic Refractory Angina Pectoris: A Randomized Trial

**DOI:** 10.3389/fcvm.2021.664433

**Published:** 2021-07-21

**Authors:** Liu Weijing, Fan Ximin, Shen Jianying, Zhu Mengyun, Fan Xuehua, Xu Yawei, Hong Liqiong

**Affiliations:** Shanghai Tenth People's Hospital, Tongji University, Shanghai, China

**Keywords:** angiogenesis, randomized trial, angina, refractory angina pectoris, cardiac shock wave therapy

## Abstract

**Background:** Cardiac shock wave therapy (CSWT) is a non-invasive new option for the treatment of chronic refractory angina pectoris (CRAP). This study aimed to evaluate the safety and efficiency of CSWT in the treatment of CRAP.

**Methods:** Eighty-seven patients with CRAP were randomly allocated into CWST group (*n* = 46) and Control group (*n* = 41). Canadian Cardiovascular Society (CCS) grade of angina pectoris, Seattle Angina Questionnaire (SAQ) score, 6-min walk test (6MWT), weekly dosage of nitroglycerin, and myocardial perfusion on D-SPECT were determined at baseline and during the follow-up period. Adverse events were also evaluated.

**Results:** CSWT was well-tolerated in the CSWT patients. CSWT significantly improved the CCS grade, SAQ score, and 6MWT (*p* < 0.05). Imaging examinations showed that the ischemic area was reduced after CSWT. However, no significant changes were observed in the Control group.

**Conclusions:** CSWT may improve the myocardial perfusion and reduce clinical symptoms without increasing adverse effects in CRAP patients. It provides a non-invasive and safe clinical therapy for CRAP patients.

**Clinical Trial registration:** www.ClinicalTrials.gov, identifier: NCT03398096.

## Introduction

Coronary artery disease (CAD) is one of the common and vital cardiovascular diseases. There are several options for the treatment of CAD, including pharmacotherapy (nitrates, beta-blockers, calcium antagonists, trimetazidine, and ivabradine), percutaneous coronary intervention (PCI), and coronary artery bypass surgery (CABG). Although interventional techniques have been widely used for the management of CAD, a few patients who are not suitable for interventional therapy suffer from chronic refractory angina pectoris (CRAP) (1, 2). It has been reported that the mortality rate of refractory angina is around 3–4% at 1 year (3, 4).

Several studies have investigated some new alternative therapeutic methods of refractory angina, including percutaneous myocardial laser revascularization, transmyocardial revascularization, and stem cell therapy. Nevertheless, these treatments are still underdeveloped, and most of them are invasive (5–7). There is evidence showing that microvascular dysfunction is one of the causes of refractory angina. This condition is likely much more common than previously reported, and many patients are experiencing microvascular angina due to infrequent assessment of microcirculatory physiology in clinical practice (8, 9).

Ultrasound-guided cardiac shock wave therapy (CSWT) is a new treatment of CAD and offers an alternative to revascularization by stimulating angiogenesis. Clinical trials have shown that CSWT can reduce the symptoms of myocardial ischemia and improve the cardiac function in patients with severe CAD (10–12). Therefore, CSWT seems to be a new non-invasive and effective therapy for chronic refractory angina.

In a variety of studies, nitroglycerin consumption, Canadian Cardiovascular Society (CCS) grade of angina pectoris, Seattle Angina Questionnaire (SAQ) scores, and New York Heart Association classification (NYHA class) are widely employed to evaluate the efficacy of CSWT (13). In only a few studies, the single photon emission computed tomography (SPECT) is used to assess the improvement of myocardial perfusion in cardiac ischemic patients. Myocardial perfusion imaging (MPI) has been well-established in the diagnosis of CAD and monitoring of therapeutic response and risk stratification in patients with known or suspected CAD. However, SPECT has limitations in the quantitative assessment. Dynamic single photon emission computed tomography (D-SPECT) imaging using multidetector SPECT systems and kinetic modeling of ^99m^Tc-teboroxime has been shown to be much better to detect the microsphere-determined blood flow than traditional SPECT. In addition, it has superior sensitivity and specificity and allows a significant reduction in the administered dose of ^99m^Tc-labeled tracers. This study was undertaken to investigate the safety and efficacy of CSWT in the treatment of CRAP.

## Patients and Methods

### Study Design and Population

The present study was registered in the clinicaltrials.gov (NCT 03398096). This was a prospective, randomized, and controlled clinical trial. The study was undertaken according to the 1975 Declaration of Helsinki and approved by the ethics committee of our hospital.

### Patients and Grouping

All patients were fully informed of the study protocol, and informed consent was obtained from each patient before the study. The inclusion criteria were as follows: (1) patients were 18–80 years old; (2) all patients were diagnosed with CAD as demonstrated by >50% stenosis on coronary angiography or multislice CT coronary angiography; (3) the patients were treated by revascularization with more than 70% of coronary artery stenosis; (4) patients had refractory angina (defined as CCS angina grading II–IV after pharmacotherapy with or without revascularization); (5) more than 1 month after acute myocardial infarction (AMI) and 1 month after PCI surgery.

The exclusion criteria were as follows: (1) patients had AMI, PCI, or CABG within 4 weeks prior to the study; (2) patients had a history of heart transplantation; (3) patients had a history of metal valve replacement; (4) patients had intracardiac thrombus; (5) patients had left ventricular ejection fraction (LVEF) <30% and unstable hemodynamics; (6) patients had arrhythmia with heart rate <40 bpm or >120 bpm; (7) patients had skin ulceration or infection at the treatment area; (8) patients had severe obstructive lung disease.

A total of 100 patients were recruited. According to the above criteria, 13 patients were excluded: four patients did not meet the inclusion criteria, eight patients declined to participate in this study, and one patient was excluded due to inconvenient transportation. Eighty-seven patients were randomly divided into CSWT group (*n* = 46) and Control group (*n* = 41). Patients in the CSWT group were treated with optimal drugs (including antiplatelet drug, statins, and antianginal drugs) + CSWT, and those in the Control group were treated with optimal drugs alone. Care providers and physicians who followed up the patients (parameters of this study) were blind to the grouping. In the CSWT group, CSWT was performed with an equipment (Modulith SLC; Storz Medical, Switzerland) according to the recommended protocol developed by the Tohoku University of Japan with respect to the shockwave output and the number of shots implemented to each spot and the protocol developed by the University of Essen, Germany (11, 14). CSWT was performed thrice weekly (first, third, and fifth days) in a course, and there was a 3-month interval between two courses. Patients received CSWT for 3 months, and a total of nine CSWTs were performed. Patients in the Control group did not receive CSWT.

### Laboratory Examinations

Blood samples of peripheral venous were collected at baseline and follow-up. Myocardial marker [creatine kinase phosphate-isozyme (CK-MB)] and hepatorenal function indexes [alanine aminotransferase (ALT), aspartate aminotransferase (AST), serum creatinine (SCr)] were measured.

### Imaging Examinations

Myocardial perfusion was evaluated using a ^99^mTc-labeled tracer SPECT (D-SPECT, Spectrum Dynamics Company) at baseline and 6 months after the first treatment in the CSWT group. One-day rest–stress method was used for adenosine load protocol with tracer injection. Summed stress score (SSS) and summed rest score (SRS) were analyzed semiquantitatively in a blind manner: SSS/SRS <4, normal; 4–8, mild abnormality; 9–13, moderately abnormal; >13, severe abnormality.

### Follow-Up

Patients were followed up at months after the first treatment by clinical examinations, quality of life assessment (Minnesota Living with Heart Failure), 6-min walk test (6MWT), echocardiography, and ^99^mTc-MIBI-labeled tracer D-SPECT. Clinical examinations included the CCS grading of angina, NYHA functional classification, SAQ scores, and nitroglycerin dose (times/week). Echocardiography was performed on a Vivid 9 (GE Vingmed, Horton, Norway). The images were stored digitally and analyzed offline by an experienced physician.

### Statistical Analysis

Statistical analyses were performed using SPSS version 15.0 (SPSS Inc., USA). Continuous data with normal distribution are expressed as mean ± standard deviation and compared using paired *t*-test at baseline and follow-up. Categorical data are expressed as frequency (n) or ratio (n/N) and compared using chi-square test. Rank data were tested using non-parametric rank sum test. A value of two-sided *p* < 0.05 was considered statistically significant.

## Results

### Clinical Characteristics

**Figure 1:**
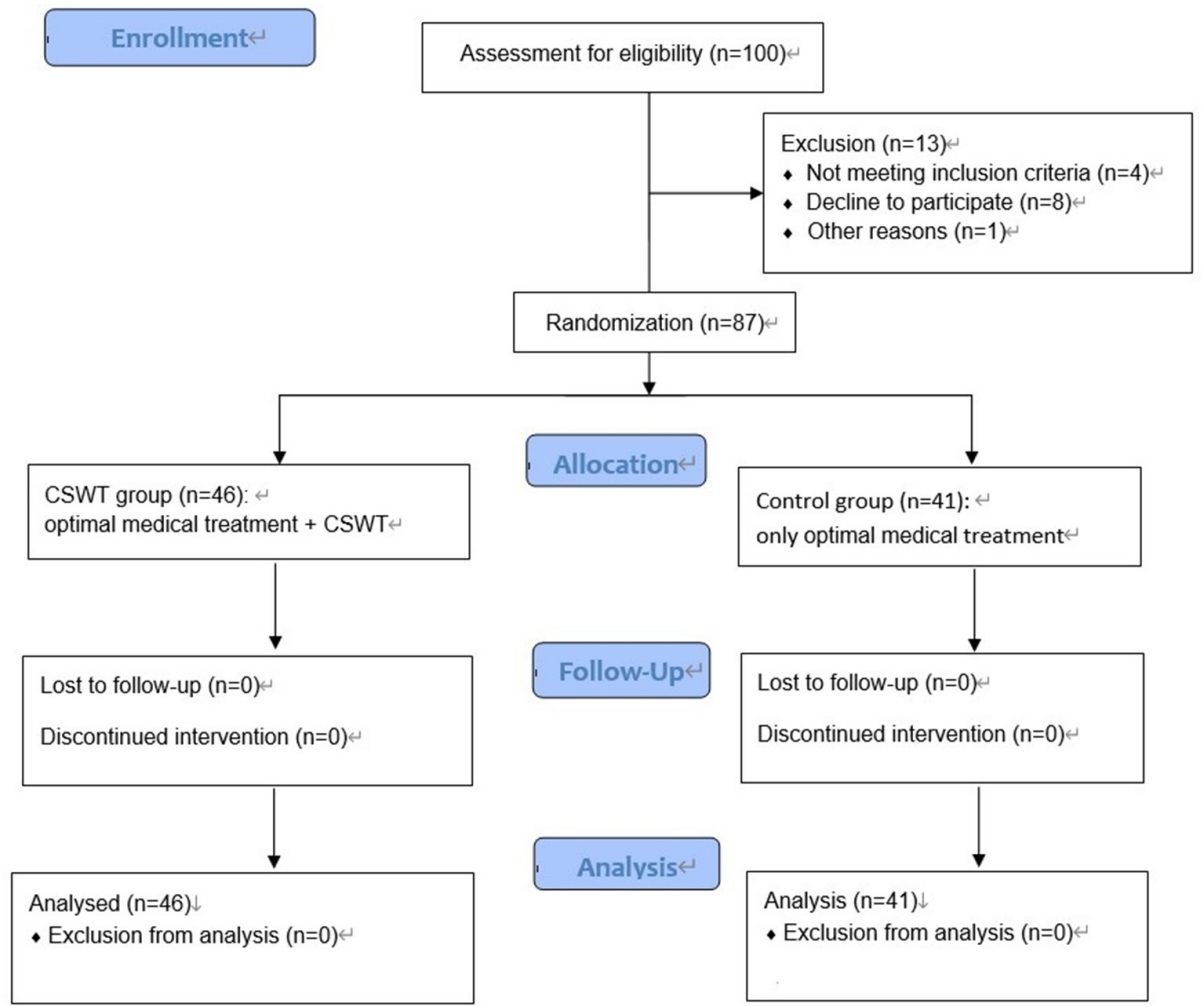
Flowchart of patient recruitment. CSWT, cardiac shock wave therapy; D-SPECT, dynamic single photon emission computed tomography.

A total of 87 patients were included in the final analysis, and the flowchart in the recruitment of these patients is shown in Figure 1. Patients' characteristics are shown in Table 1. There were 46 patients in the CSWT group and 41 patients in the Control group. The average age was 68.1 ± 6.7 years in the CSWT group and 68.9 ± 6.6 years in the control group, with no significant difference between the two groups. The average body mass index (BMI) in the CSWT group and control group was 24.7 ± 3.8 and 24.9 ± 3.7, respectively, with no significant difference. There were no significant differences in the history of hypertension, diabetes, or hypercholesterolemia between the CSWT group and Control group (*p* > 0.05). There were no significant differences in therapeutic drugs between the CSWT group and Control group (*p* > 0.05). No patients were lost in the follow-up.

**Table 1:** Characteristics of patients in two groups.

	**CWST group**	**Control group**	***P***
	**(*n* = 46)**	**(*n* = 41)**	
Age, years	68.1 ± 6.7	68.9 ± 6.6	0.507
Male, *n* (%)	32 (70)	29 (71)	0.547
BMI, kg/m^2^	24.7 ± 3.8	24.9 ± 3.7	0.688
Smoking, *n* (%)	17 (37)	14 (34)	0.826
Hypertension, *n* (%)	27 (59)	23 (56)	0.831
Diabetes, *n* (%)	24 (52)	23 (56)	0.830
Hypercholesterolemia, *n* (%)	20 (43)	18 (44)	0.570
Aspirin, *n* (%)	41 (89)	36 (88)	0.554
Calcium channel blockers, *n* (%)	15 (33)	14 (34)	0.530
ß Blockers, *n* (%)	24 (52)	23 (56)	0.521
ACEI/ARB, *n* (%)	22 (48)	18 (44)	0.830

*Data are shown as Mean ± standard deviation. CSWT, cardiac shock wave therapy; BMI, body mass index; ACEI, angiotensin converting enzyme inhibitor; ARB, angiotensin receptor blocker*.

### Biochemical Parameters

The myocardial ischemia markers (CK-MB), hepatorenal function (AST and ALT), and renal function (SCr) were detected at baseline and 6 months after treatment. Results showed no significant differences in these parameters. It is indicated that the procedure of CSWT is safe and did not result in damage to the myocardium (Table 2).

**Table 2:** Biochemical parameters at baseline and follow-up.

**Group**	***n***	**Time**	**ALT (U/L)**	**AST (U/L)**	**CK-MB (ng/ml)**	**SCr (μmol/L)**
CSWT	46	Baseline	28.9 ± 7.9	35.4 ± 7.3	27.8 ± 10.6	67.4 ± 28.4
		Follow-up	31.6 ± 8.5	28.6 ± 8.7	26.9 ± 12.5	78.4 ± 26.4
Control	41	Baseline	26.4 ± 8.2	27.9 ± 6.5	24.5 ± 8.9	59.2 ± 28.8
		Follow-up	30.8 ± 8.6	26.4 ± 8.3	26.7 ± 9.4	66.8 ± 24.3

*Data are shown as mean ± standard deviation. There were not significant differences between CSWT group and Control group at baseline and follow-up in these parameters (P > 0.05). CSWT, cardiac shock wave therapy; ALT, alanine aminotransferase; AST, aspartate aminotransferase; CK-MB, creatine kinase phosphate-isozyme; SCr, serum creatinine*.

### Clinical Parameters

There were significant differences in the majority of clinical parameters between the two groups at 6 months. The clinical symptoms (chest tightness and chest pain) were all improved in the CSWT group. The symptoms were evaluated by CCS class scores, SAQ scores, and 6MWT. At baseline, there were no significant differences in the CCS score, SAQ score, and results from 6MWT between the two groups (*p* > 0.05). However, 6 months after treatment, these parameters were significantly improved in the CSWT group as compared to the Control group (*p* < 0.05). The average CCS class scores were 2.90 ± 0.57 at baseline and 2.10 ± 0.32 at 6 months in the CSWT group. The average SAQ scores were 63.3 ± 15.3 at baseline and 75.6 ± 10.5 at 6 months in the CSWT group. The result of 6MWT was 331.7 ± 62.3 and 403.1 ± 61.2 at baseline and 6 months, respectively, in the CSWT group. Furthermore, nitrate consumption in the CSWT group decreased as compared to the Control group (0.90 ± 0.68 vs. 1.60 ± 0.52, *p* = 0.01) (Table 3).

**Table 3:** CCS, SAQ, 6MWT and nitroglycerin consumption at baseline and follow-up.

	**Parameters**	**CSWT**	**Control**	***P***
		**(*n* = 46)**	**(*n* = 41)**	
Baseline	CCS (grade)	2.90 ± 0.57	2.80 ± 0.79	0.15
	SAQ (score)	63.3 ± 15.3	65.6 ± 14.6	0.53
	6MWT (m)	331.7 ± 62.3	319.3 ± 69.3	0.45
	Nitrate consumption (times/week)	2.40 ± 1.26	2.10 ± 1.02	0.45
Follow-up	CCS (grade)	2.10 ± 0.32	2.90 ± 0.57	0.002
	SAQ (score)	75.6 ± 10.5	67.3 ± 13.3	0.03
	6MWT (m)	403.1 ± 61.2	336.7 ± 71.1	0.0001
	Nitrate consumption (times/week)	0.90 ± 0.68	1.60 ± 0.52	0.01

*Data are shown as mean ± standard deviation. CSWT, cardiac shock wave therapy; CCS, Canadian Cardiovascular Society grade of angina pectoris; SAQ, Seattle Angina Questionnaire score; 6MWT, 6-minute walking distance test*.

### Imaging Examination

**Figure 2:**
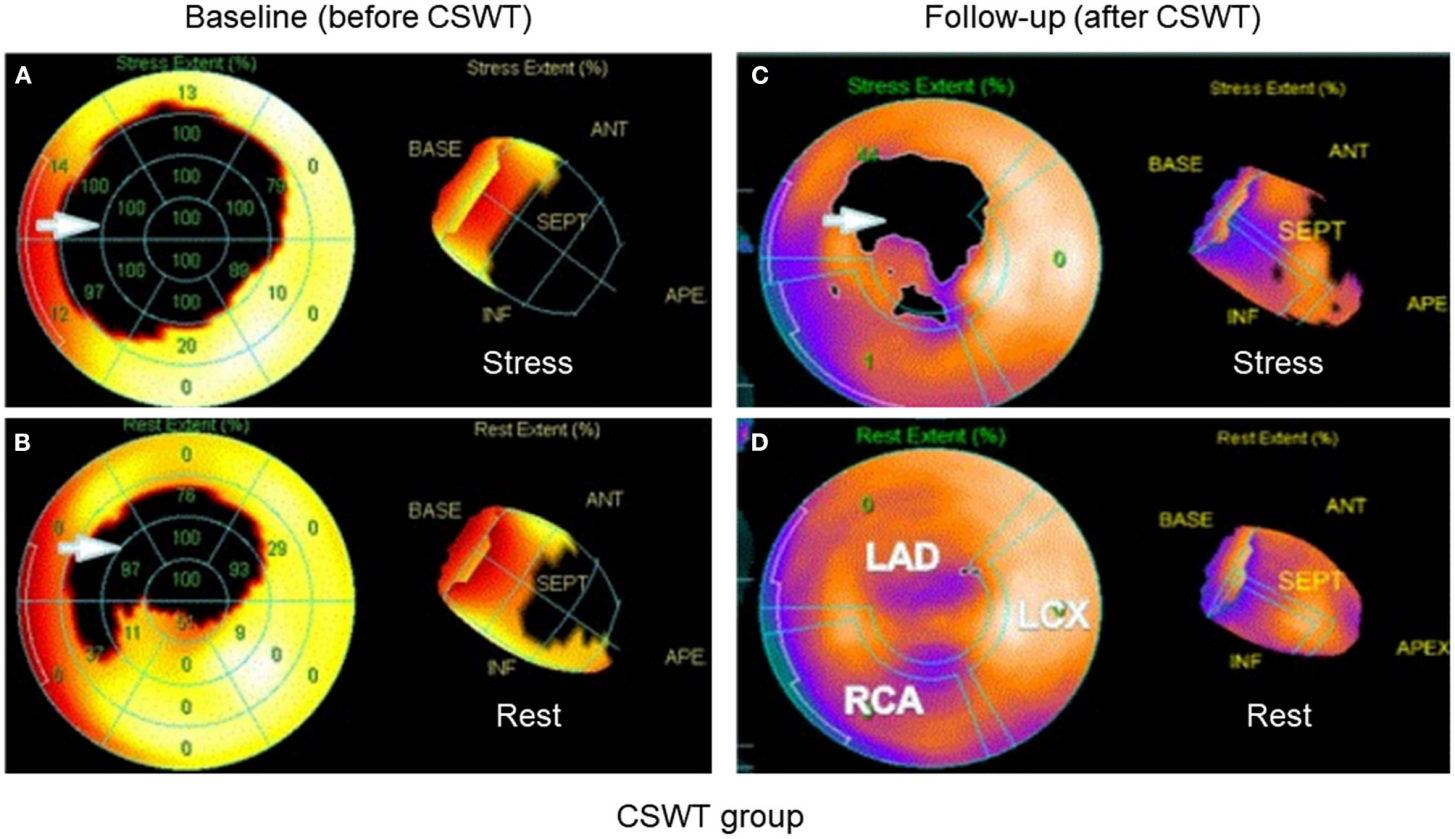
D-SPECT of a patient. After CSWT, the ischemic myocardium at rest and stress procedure was significantly reduced at 6 months after treatment as compared to that before CSWT. **(A)** Before treatment at stress procedure. **(B)** Before treatment at rest procedure. **(C)** After treatment at stress procedure. **(D)** After treatment at rest procedure. Arrow: ischemic area. CSWT, cardiac shock wave therapy; D-SPECT, dynamic single photon emission computed tomography; LAD, left anterior descending artery; LCX, left circumflex coronary artery; RCA, right coronary artery.

D-SPECT showed that the ischemic area was significantly reduced on the stress procedure at 6 months after CSWT in the CSWT group as compared with that at baseline. In addition, CSWT improved the myocardial perfusion in the treated area as evaluated by D-SPECT in the adenosine stress protocol. In the CSWT group and the Control group, the SSS was 16.27 ± 7.64 and 16.45 ± 5.05, respectively, and the SRS was 7.17 ± 2.62 and 7.06 ± 3.86, respectively, at baseline. However, both SSS and SRS were reduced in the CSWT group at 6 months. The SSS was 13.64 ± 6.69 and 16.82 ± 6.83 in the CSWT group and Control group, respectively (*p* > 0.05). The SRS was 6.73 ± 1.86 and 7.08 ± 2.64 in the CSWT group and control group, respectively, at 6 months (*p* > 0.05). The SSS remained unchanged after CSWT in the CSWT group (*p* < 0.05) (Table 4, Figure 2).

**Table 4:** Imagine Findings at baseline and follow up.

	**Parameters**	**CSWT (*n* = 46)**	**Control (*n* = 41)**	***P***
Baseline	SRS (score)	7.17 ± 2.62	7.06 ± 3.86	0.326
	SSS (score)	16.27 ± 7.64^*^	16.45 ± 5.05	0.781
	LVDd	45.17 ± 8.03	46.90 ± 8.47	0.628
	LVEF	48.80 ± 6.47	47.83 ± 7.78	0.758
Follow up	SRS (score)	6.73 ± 1.86	7.08 ± 2.64	0.069
	SSS (score)	13.64 ± 6.69^*^	16.82 ± 6.83	0.057
	LVDd	48.60 ± 5.62	48.82 ± 6.27	0.813
	LVEF	53.75 ± 5.85	48.50 ± 6.96	0.069

*Data are shown as mean ± standard deviation. CSWT, cardiac shock wave therapy; SSS, Summed stress score; SRS, summed rest score; LVEF, left ventricular ejection fraction*.

* *P < 0.05, Baseline vs. Follow-up in the CSWT group*.

Echocardiography was performed on a Vivid 9 (GE Vingmed, Horton, Norway). Echocardiography was done to illustrate the left ventricular end-diastolic dimension (LVDd) and left ventricular ejection fraction (LVEF). As shown in Table 4, the LVEF was similar at baseline between the two groups, and it remained unchanged after treatment (6 months). The LVDd was also comparable between the two groups at baseline, and it remained unchanged after treatment (6 months). This reflected that LV function had no signs of deleterious LV remodeling.

### Adverse Effects

CSWT was well-tolerated in all the subjects. No complications or adverse effects were noted in the patients of the two groups.

## Discussion

In the present study, results showed that the CCS grade of angina, SAQ score, 6MWT, and nitrate consumption were all improved in the CSWT group. Furthermore, the ischemic area on D-SPECT was also reduced after CSWT. However, there were no significant changes in the outcomes in the Control group. These results suggest that CSWT can achieve a favorable clinical efficacy as well as a better quality of life for patients with CRAP.

Increasing clinical studies on CSWT have been published since 1999. In the majority of CSWT-related studies, results indicate that nitroglycerin consumption is reduced; angina frequency is decreased; CCS grade, SAQ score, and NYHA class score are improved; and exercise capacity is increased significantly after CSWT (13). Myocardial perfusion can be assessed by conventional SPECT, but the low sensitivity and low temporal resolution of conventional SPECT limit its wide use in clinical practice further assessment (15, 16). In the present study, D-SPECT was employed to evaluate myocardial perfusion. As a new technique, D-SPECT has better sensitivity and specificity and can provide more accurate professional information.

CSWT is suitable for patients with refractory CAD, CCS angina grade of III/IV, nonresponse to two or three anti-anginal drugs within at least 8 weeks, recurrent angina pectoris after PCI/CABG, or severe CAD unsuitable for interventional revascularization. In 2003, at the European Society of Cardiology, Nishida et al. (17) for the first time reported the therapeutic effect of CSWT in animal models of CAD and CAD patients. CSWT has been used in clinical trials and scientific studies in nine countries including Germany, Japan, Switzerland, Italy, and China and has achieved good clinical efficacy.

The mechanism underlying the therapeutic effects of CSWT is complex. Yip et al. (18) investigated the effect of shock wave on the femoral bone of adult male Sprague–Dawley rats and found that the shock wave could induce the formation of vascular endothelial growth factor (VEGF) and increase the CD31-positive cells (an endothelial phenotype), which accelerated the differentiation of bone marrow cells into endothelial cells (16). In addition, CSWT may serve as an alternative to revascularization by stimulating angiogenesis in the ischemic myocardium, which ameliorates myocardial ischemia (17, 19, 20). Studies have shown that shock wave may also affect the expression of chemokines and matrix metalloproteinases to confer anti-inflammatory effects, activate Ras, stimulate nitric oxide (NO) synthesis, and upregulate VEGF and its receptor, *fms*-like tyrosine kinase (Flt)-1 (17, 19, 21–25). Whether all these effects contribute to the improvement of cardiac function is still unclear. However, it has been confirmed that CSWT can stimulate angiogenesis in the ischemic myocardium by upregulating VEGF expression. VEGF is an angiogenic factor. Nishida et al. (17) found that CSWT could upregulate mRNA expression of VEGF both *in vitro* and *in vivo*. In a porcine model of chronic myocardial ischemia, the LVEF, wall thickening fraction, and regional myocardial blood flow of the ischemic region were completely improved significantly in 4 weeks after shock wave treatment as compared to control animals.

In the CSWT, the patient is asked to lie in a supine position and relax, and the electrocardiogram, blood pressure, and blood oxygen saturation are monitored. Several studies have indicated that three CSWT sessions within 1 month may achieve the same efficacy as 3-month CSWT (10, 26, 27). This finding is encouraging, but whether a shorter term or less frequent CSWT can achieve a similar therapeutic effect is still unclear, and more clinical studies are needed to elucidate this issue.

One of the strengths in the present study is the use of D-SPECT in the evaluation of ischemic myocardium. Through D-SPECT, the cardiac perfusion and cardiac function can be objectively assessed by radionuclide myocardial perfusion imaging. Great progress has been achieved in the D-SPECT due to the development of imaging equipment. As compared to the traditional SPECT with sodium iodide (NaI) crystals, the latest D-SPECT uses the most advanced all-digital, cadmium zinc telluride (CZT) solid-state detector, which increases the sensitivity by 10 times, the resolution by two times, and the scanning speed by 10 times. In our study, D-SPECT was employed to evaluate the cardiac perfusion in the ischemic area, which may be helpful for the assessment of the therapeutic efficacy of CSWT. In previous studies, traditional SPECT was mainly used to determine the improvement of myocardial perfusion in myocardial ischemia patients. Hence, the use of D-SPECT in our study is a novelty.

The combination of CSWT and D-SPECT in clinical practice may benefit patients with CRAP because CSWT can improve ischemic symptoms and myocardial perfusion in patients non-responsive to interventional therapy, and D-SPECT is a safe and simple examination that can objectively and reliably assess the ischemic area after CSWT.

However, there were limitations in the present study. First, only short-term follow-up was administered in our study, and the long-term efficacy of CSWT should be further confirmed. Second, treadmill exercise test can be employed to evaluate the exercise tolerance of patients. It is a non-invasive examination and can also be used to assess the clinical improvement of patients after CSWT.

## Conclusions

The present study indicates that CSWT can improve CCS grade of angina, SAQ score, 6MWT results, and nitrate consumption in patients with CRAP. Furthermore, D-SPECT shows that the myocardial ischemic area is reduced after CSWT. The significant improvement of angina symptoms may be associated with the reduction of ischemic myocardium. Therefore, CSWT is a non-invasive, safe, and easy-to-use treatment for patients with CRAP and may serve as a good alternative for the treatment of CRAP, achieving favorable clinical therapeutic efficacy and better quality of life.

## Data Availability Statement

The raw data supporting the conclusions of this article will be made available by the authors, without undue reservation.

## Ethics Statement

The studies involving human participants were reviewed and approved by Shanghai Shen Kang Hospital Development Center. The patients/participants provided their written informed consent to participate in this study. Written informed consent was obtained from the individual(s) for the publication of any potentially identifiable images or data included in this article.

## Author Contributions

LW: design, definition of intellectual content, literature search, and statistical analysis. FXi: literature search, clinical studies, data acquisition, and manuscript preparation and editing. SJ: data acquisition and literature search. ZM and FXu: data acquisition. XY: concept, design, definition of intellectual content, manuscript review, and gain the grant. All authors contributed to the article and approved the submitted version.

## Conflict of Interest

The authors declare that the research was conducted in the absence of any commercial or financial relationships that could be construed as a potential conflict of interest.
